# Improvement of vibration and acoustic properties of woven jute/polyester composites by surface modification of fibers with various chemicals

**DOI:** 10.1038/s41598-025-99185-4

**Published:** 2025-06-04

**Authors:** S. Senthilrajan, N. Venkateshwaran, S. O. Ismail, Rajini Nagarajan, Nadir Ayrilmis, Kumar Krishnan, Faruq Mohammad, Hamad A. Al-Lohedan

**Affiliations:** 1 Department of Robotics and Automation, Rajalakshmi Engineering College, Chennai, India; 2 Centre for Digital Manufacturing, Department of Mechanical Engineering, Rajalakshmi Engineering College, Chennai, India; 3 Centre for Engineering Research, Department of Engineering, University of Hertfordshire, Hatfield, Hertfordshire, England AL10 9AB UK; 4 Department of Mechanical Engineering, Kalasalingam Academy of Research and Education, Krishnankoil, Tamilnadu India; 5 Department of Wood Mechanics and Technology, Faculty of Forestry, Istanbul University-Cerrahpasa, Sariyer, 34473 Istanbul, Turkey; 6 INTI International University, Persiaran Perdana BBN, 71800 Nilai, Negeri Sembilan Malaysia; 7 Department of Chemistry, College of Science, King Saud University, P.O. Box 2455, 11451 Riyadh, Kingdom of Saudi Arabia

**Keywords:** Jute fiber, Vibration damping, Sound absorption, Surface modification, Mechanical properties, Green product, Engineering, Materials science

## Abstract

In response to the growing demand for lightweight and sustainable materials, the integration of natural fibers into polymer matrix composites has become very important. To improve the compatibility between hydrophilic natural fibers and matrices, surface modification has proven to be a crucial step. Therefore, this study advanced this technique by modifying the surfaces of woven jute mats with 1% of sodium hydroxide (NaOH), chromium sulphate (Cr_2_SO_4_), and sodium bicarbonate (NaHCO_3_). The composite, containing 56% by volume of the fibers, was produced by compression moulding. Extensive testing was carried out, including three-point bending, free vibration mode and acoustic analysis. The Brüel and Kjær two-microphone impedance tube with a frequency range of 25–6400 Hz was used. Various properties such as bending strength, vibration behaviour, damping and sound absorption were evaluated. It was comparatively evident that the NaHCO_3_-treated composite samples exhibited the highest natural frequency of 61.04 Hz and the highest sound absorption coefficient of 0.67 at about 2000 Hz, which was 69% higher than that of the untreated composite samples and about 29–72% higher than that of other treated counterparts. In addition, other test results of the surface modified composites were better than the untreated counterparts. There was good agreement between the experimental data and the results obtained from the theoretical models, which is another significant contribution to the field of composite technology.

## Introduction

Due to environmental security and preservation of environment, industries have concentrated on the use of the natural materials. Nowadays, natural fibers are significantly replacing traditional synthetic fibers, as a reinforcement in polymer matrix composites^1^. Natural fibers are biodegradable, lightweight, low cost, high specific strength materials. They possess better properties when compared with synthetic counterparts (glass and carbon). Recycling and renewing natural plant sources, including bast, seed, leaf, wood and fruit, are driving the development of environmentally acceptable green materials. Compared to the architecture of other natural fibers, such as hemp, flax, sisal, coir, bamboo, and aloe vera, jute fiber is an essential fiber consisting of bundled ultimate cells with spirally oriented microfibrils connected together^2^. High specific strength and modulus, availability, low cost, lightweight, recyclability, biodegradability, lack of health risks and non-abrasive nature are the key benefits of the natural fiber-reinforced composites^3^. A microcrystalline structure with high order of crystalline regions comprises the majority of the cellulose. In general, cellulose concentration and microfibril angle have a significant impact on the mechanical properties of the resulting composites, which results to an increased stiffness. The jute fiber also contains hemicellulose, lignin, pectin, waxy materials and water-soluble compounds^4^.

Even though, natural fibers have low wettability and a high moisture absorption rate due to their structural characteristics, there is an insufficient adhesion between the fibers and polymer matrix. This causes debonding with usage and ageing^5^. A major challenge in using natural fibers as a reinforcement in hydrophobic polymer matrices is the inherent poor compatibility between the fibers and the matrix. This mismatch leads to limitations in stress transfer from the matrix to the fibers, which are exacerbated by dimensional variations in the fibers and can cause microcracks in the composites. Consequently, the mechanical properties of their composites are compromised. To improve the adhesion between the fibers and the matrix, the surface of the fibers is usually modified by physical and chemical methods, among other means. Both approaches aim to reduce moisture absorption, while causing changes to the fiber surface. Physical treatments, such as corona, plasma and electron beam irradiation treatment lead to changes in the surface properties of natural fibers. These changes, in turn, strengthen the mechanical bond between fibers and matrix without changing the chemical composition of the fibers^4^. Traditionally, the primary purpose of chemically treatment of natural fibers is to remove unwanted non-cellulosic substances in order to enhance the performance of fiber-reinforced composites. For example, alkali, silane and coupling agent called maleic anhydride grafted polypropylene have been used to treat the surface of fibers in various ways and improve the adhesion between fibers and matrix^4^. Several studies have reported increase in the interfacial strength and mechanical properties, using some binders, such as latex, Polyvinyl alcohol (PVA), chitosan, among others. In addition, PVA treatment increased the ability of acoustic characteristics^6^. In addition to vibration damping, structural engineers must consider the acoustic characteristics of the material to reduce noise.

The combination of sound absorption coefficient and transmission loss is an important factor^7^. Senthilrajan et al.^8^ studied the ageing effect of vibration characteristics of jute/polyester composites. It was concluded from their studies that ageing of normal water and seawater had greater influence on the vibration behavior of composites. Vinayakapatil et al.^9^ investigated into the vibration characteristics of jute fiber/polyester composite plate. In their investigation, they studied different parameters, including fiber shape, orientations and coupling agent weight percentage (wt%) of jute fiber. From the results obtained, it was reported that the 6% of coupling agent had better damping ratio than other type of the combinations. Ramakrishnan et al.^10^ reported free vibration and dynamic mechanical characteristics of chemically treated jute fiber/nano-clay composites. NaOH treatment and addition of the nano-clay with different wt% were considered to improve the performance of the composites. It was concluded that the 5 wt% nano-clay incorporation and 5 wt% treated composite was recommended for structural applications. Rajeshkumar et al.^11^ studied the composition of sustainable *Phoenix *sp. fibers and nanoclays to improve the free vibration properties of epoxy polymers for machine tool applications. Different parameters, such as fiber length, fiber volume fraction, concentration of NaOH treatment and wt% of nano-clay were considered. The result established that both surface modification and presence of fillers supported better vibrational characteristics. Moving forward, Prabhakaran et al.^12^ investigated into the acoustics and vibration damping properties of flax fiber reinforced composites, using different layering arrangements. They concluded that the flax fiber composite recorded better mechanical, vibration and acoustic behaviors than glass fiber composites. Munde et al.^13^ reported that the sisal fiber-reinforced polypropylene composite vibration damping and acoustic properties. They used the sisal fiber as a reinforcement with different fiber loading in wt% to polypropylene. The result reveals that the higher weight percentage of sisal fiber to the matrix are enhancing better vibration and acoustics characteristics. Senthilrajan et al.^14^ studied the influence of different jute fiber parameters on vibration and acoustic characteristics of jute fiber polyester composite, using different fiber parameters and wt%. It was concluded that the fiber parameters had greater influence on vibration and acoustic characteristics of the composites. Furthermore, Senthilrajan et al.^15^ reported effects of different fiber lengths and surface modification on acoustic characteristics of jute fiber. The result revealed that the fiber length recorded greater influence on acoustic characteristics, while surface modified fibers had lower absorption coefficient than the untreated fibers. In addition, the experimental results had a good agreement with theoretical results. Saygilim et al.^16^ investigated into the acoustic and mechanical properties of jute and luffa fiber/epoxy composites. The result showed that both types of composites exhibited better acoustic and elastic moduli. Hence, it was concluded that the luffa and jute fibers can be used to produce the samples with high damping and stiffness. Samaei et al.^17^ reported the effect of alkaline treatment on acoustic, mechanical and thermal behaviors of kenaf fiber. The investigation involved the use of NaOH with different concentrations and immersion times for surface modification. It was reported that both increasing concentration and immersion time improved the composites.

Based on the above research study, it can be concluded that the key determinant of the properties of composite materials is the interfacial adhesion between fiber and matrix. To improve the adhesion, modification of the inherent hydrophilic properties of the fiber is crucial. Among the various techniques available, chemical treatment stands out as a cost-efficient and effective method. Considering the pursuit of sustainability, natural fibers represent a promising resource. Therefore, this research addresses the modification of jute fiber surfaces using different chemicals at equivalent concentrations. Subsequent analysis on their resulting effects on vibration and acoustic properties was comprehensively reported.

## Materials and methods

### Modification of jute fibers and composite preparation

The jute fiber woven mat of plain weave pattern was obtained from the National Jute Board in Chennai, India. For the surface modification, the woven jute mat was subjected to various surface treatments such as alkali (NaOH), NAHCO_3_ and Cr_2_SO_4_ chemicals at 1 wt% each at room temperature. The fibers were separately dipped in the solutions for 30 min and then washed with running water to remove the excess amount of chemicals and the treated fiber was dried in sunlight for two days. The polyester resin, with an accelerator/cobalt naphthalene and the catalyst/methyl ethyl ketone peroxide (MEKP) were purchased from local dealers and used. The mould was prepared for laminate with dimensions of 300 mm × 300 mm × 3 mm. In the current study, a 300 mm × 300 mm woven jute mat was used as the reinforcement material. The polyester resin mixture was cured using 1% of a catalyst called methyl ethyl ketone peroxide (MEKP) and an accelerator called cobalt naphthalene. The composite laminates were produced by compression molding at room temperature. The laminates were produced by compression molding at room temperature. After curing for 4 h, the composite laminates were removed from their moulds, and the samples were cut in accordance with ASTM standards for flexural, free vibration and sound absorption tests. THE codes of the composite samples and their nomenclatures are given in Table 1.

**Table 1:** Matrix and other four woven jute mat/polyester composite samples used and their nomenclatures.

S/no	Sample	Nomenclature
1	Polyester resin	PR
2	Untreated	UT
3	NaOH treated	SM1
4	NaHCO_3_ treated	SM2
5	Cr_2_SO_4_ treated	SM3

### Test methods

**Fig. 1:**
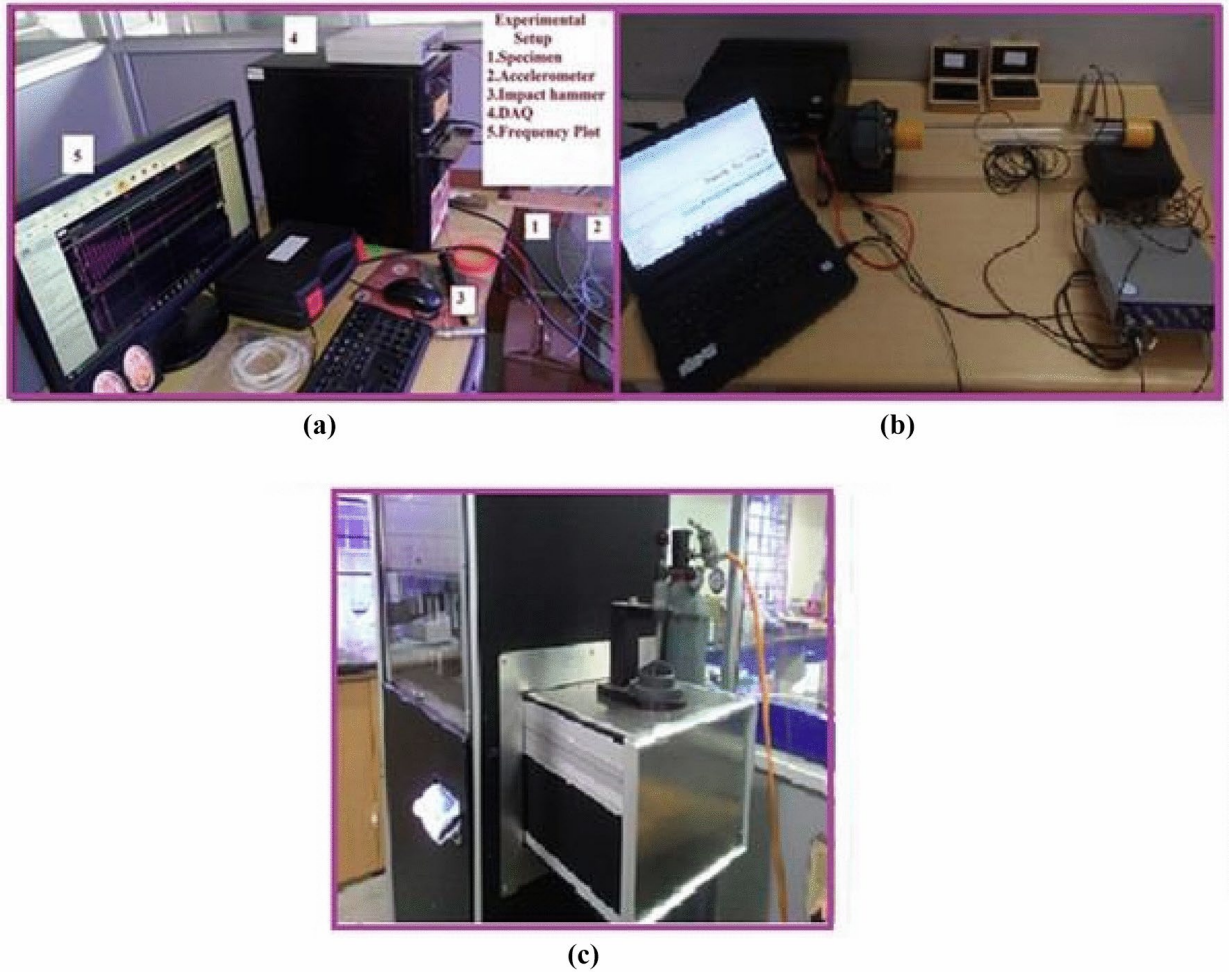
(**a**) Free vibration test set-up, (**b**) impedance tube and (**c**) air flow resistivity apparatus.

The computerised universal testing machine was used to conduct three-point flexural test with a specimen size of 127 mm × 12.7 mm × 3.2 mm at a testing speed of 2 mm/min in accordance with ASTMD-790. In this free vibration test (FVT), rectangular samples with the dimension of 250 mm × 25 mm × 3 mm were prepared and used. The test was carried out in a cantilever mode; the displacement of the samples was measured by the accelerometer 8778A500sp, which was attached to the free end of each sample. The sample was excited with a rubber impact hammer (Model 1H-01). The output data of the accelerometer and impact hammer were stored, using DEWSoft 7.1.1 software. The amplitude *versus* frequency and acceleration *versus* time plots were generated and used to determine the natural frequency and damping ratio (ζ), using Eq. (1).

1 $$\upxi =\frac{1}{2\pi j}\text{ln}\frac{Xi}{Xi+j}$$

where x_i_ is the peak acceleration of the ith peak and x_i+j_ is the peak acceleration of the peak j cycles after ith peak. The experimental set-up for the free vibration test and test samples are shown in Fig. 1.

### Theoretical modal analysis

Vibration testing was employed to study the dynamic behavior of the surfaced jute/polyester composite. The damping factor and modal characteristics of the composite were determined through this test. The Euler–Bernoulli equation was used to determine the theoretical natural frequency, ω_1_ of a cantilever beam, as expressed in Eq. (2).

2 $${\upomega _1} = {\left( {\upbeta {\text{L}}} \right)^2}\sqrt {\frac{{{\text{EI}}}}{{\rho A{L^4}}}} \;{\text{rad}}/\sec$$

where E = modulus of the beam in MPa, I = moment of Inertia in mm^4^, ρ = density of composites in kg/mm^3^, A = cross-sectional area of the cantilever beam in mm^2^, L = length of the beam in mm and β was determined from the boundary conditions.

### Sound absorption study

According to the ASTM E1050 standard, the sound absorption experiment was conducted, based on a two-microphone transfer-function technique within the frequency range of 63 to 6300 Hz. Four samples were tested, each has a diameter and thickness of 100 and 29 mm, respectively.

### Air flow resistivity

The flow resistivity (σ) of jute fiber (JFL4) was measured, using the ASTMC522 standard. Five samples were considered and their results were averaged. The measurements of the pressure difference were maintained between 0.1 and 250 Pa. From this air flow apparatus, the specific airflow resistivity was measured in the range of 100 to 12,000 Pa s/m^2^.

### Theoretical predication of sound absorption properties of natural fibers

The parameters used for the theoretical predicted parameters of jute fiber composites are given in Table 2.

**Table 2:** Various parameters used for theoretical calculation of jute fiber.

Fiber diameter (µm)	Fiber bulk density (g/cm^3^)	Flow resistivity (Pa s/m^2^)
50–60	1.3	12,000

Delany-Bazley Model and Garai Pompoli Model equations were used to calculate the theoretical sound absorption, as shown in Eqs. (3–6).

Delany-Bazley Model:

3 $${\varvec{Z}}\text{c}=\uprho_0 {\text{C}} \lceil1+0.0571{\left(\frac{\uprho 0\text{ f}}{\upsigma }\right)}^{-0.754}-\text{j }0.087{\left(\frac{\uprho 0\text{ f}}{\upsigma }\right)}^{-0.732}\rceil$$

4 $${\varvec{K}}\text{c}= \frac{\upomega }{\text{C}}\lceil1+0.0978{\left(\frac{\uprho 0\text{ f}}{\upsigma }\right)}^{-0.7}-\text{j }0.189{\left(\frac{\uprho 0\text{ f}}{\upsigma }\right)}^{-0.595}\rceil$$

GaraiPompoli Model

5 $${\varvec{Z}}\text{c}=\uprho_{0}\text{C}\lceil1+0.078{\left(\frac{\uprho 0\text{ f}}{\upsigma }\right)}^{-0.623}-\text{j }0.074{\left(\frac{\uprho 0\text{ f}}{\upsigma }\right)}^{-0.660}\rceil$$

6 $${\varvec{K}}\text{c}=\uprho _{0}\text{C}\lceil1+0.121{\left(\frac{\uprho 0\text{ f}}{\upsigma }\right)}^{-0.53}-\text{j }0.159{\left(\frac{\uprho 0\text{ f}}{\upsigma }\right)}^{-0.571}\rceil$$

where Zc represents acoustic impendence, Kc denotes propagation constant, $\uprho$_0_ and C denote the density and speed of the air media respectively, while σ and f stand for the flow resistivity and frequency (ω = 2πf), respectively.

### Fractography study

Interfacial bonding of the fiber and matrix was examined with aid of ZEISS SUPRA55 scanning electron microscope (SEM) with an accelerating voltage of 5.00 kV.

### Wettability study

The contact angle is a commonly used parameter to determine the wettability of a solid substrate by a liquid. The contact angle meter DMe-211 Kyowa Interface Japan was used to determine the contact angle.

## Results and discussion

### Flexural modulus

**Fig. 2:**
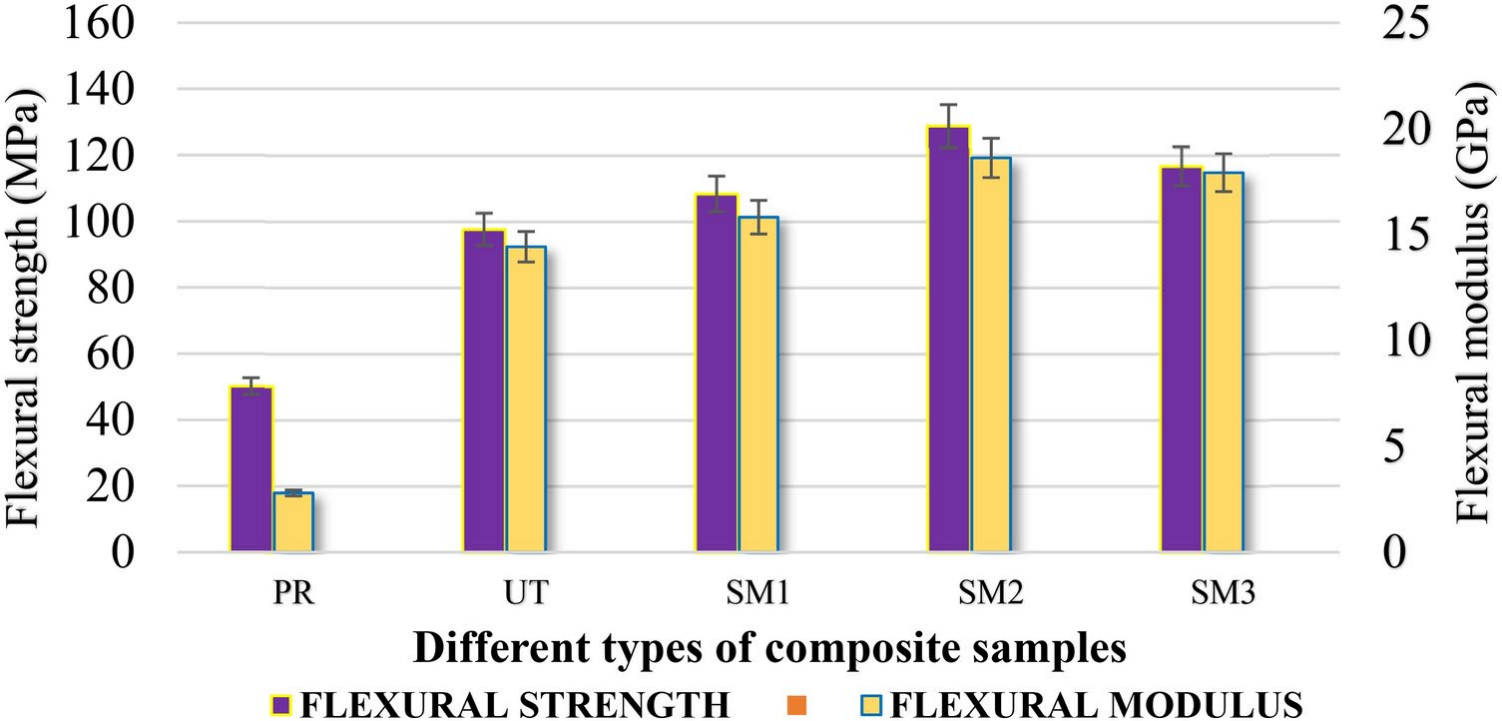
Flexural properties of the composite samples.

The flexural moduli of different types of surface modified by NaOH (SM1), NaHCO_3_ (SM2) and Cr_2_SO_4_ (SM3) composites were obtained, using three-point bending mode and were compared with the untreated composite samples, according to the ASTMD-790 standard. The performance comparison of various composites is shown in Fig. 2, which revealed an interesting trend. The composite sample SM2 exhibited best performance, having a remarkable flexural strength of 116.72 MPa. In comparison with the untreated composite counterparts, this achievement marked a significant advancement. Additionally, when compared with the untreated composites, the composite sample SM2 recorded modulus value of 18.62 GPa. This was a noteworthy improvement of almost 23%. This increase in modulus highlighted its increased stiffness and structural integrity provided by the treatment, highlighting its effectiveness. The experimentally obtained modulus values were later used to predict the natural frequency of the composite in analytical method.

**Fig. 3:**
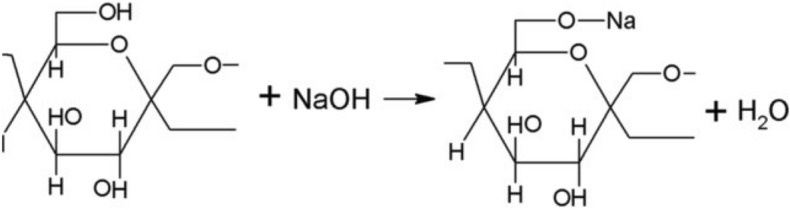
Chemical reaction of jute fibers with sodium hydroxide^20^.

The flexural properties was increased, due to the removal of waxes, gums and cement components (Fig. 2). In addition, a study reported that chemical treatment increased the cellulose content in the fibers^18^. The chemical reaction between sodium hydroxide and cellulose in jute fiber is given in Fig. 3.

Specific changes caused by alkali treatment (sodium hydroxide) of jute fibers include swelling of the fibers, removal of non-cellulosic materials, such as lignin, increased accessibility of hydroxyl groups, and changes in the surface energy and polarity of the fiber. These changes increased the affinity of the fibers for certain chemicals and improved their compatibility with polymer matrices when used in composites^19^. As well known, an aqueous solution of sodium bicarbonate is mildly alkaline, due to the formation of carbonic acid and hydroxide ion, as shown in Eqs. (7) and (8):

7 $${\text{NaHCO}}_{{3}} + {\text{ H}}_{{2}} {\text{O }} \to {\text{Na}}^{ + } + {\text{ HCO}}_{{3}}$$

8 $${\text{HCO}}_{{3}} + {\text{ H}}_{{2}} {\text{O}} \rightleftharpoons {\text{H}}_{{2}} {\text{CO}}_{{3}} + {\text{ OH}}$$

**Fig. 4:**
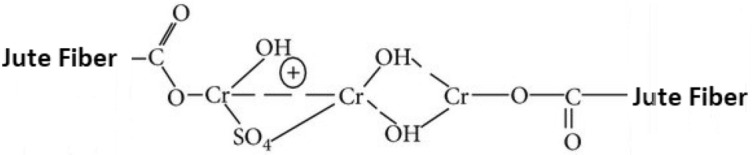
Chemical reaction of jute fiber with sodium bi-carbonate^30^.

The alkaline nature of sodium bicarbonate contributed to alteration in chemistry of the surface of the fiber. This might lead to changes in the accessibility of hydroxyl groups and other functional sites on the jute fibers (Fig. 4), affecting their affinity for moisture^20^. In addition, this interaction led to the formation of covalent bonds between the cellulose molecules and the chromium ions. The resulting complexes are usually water-insoluble and caused a crosslinking within the cellulose structure. Cellulose-chromium complexes improve the durability, flame resistance and moisture resistance of the fiber and its composites. The addition of chromium sulphate to cellulose modified its properties, making it more resistant to moisture, heat and chemicals. This crosslinking improved the dimensional stability and mechanical strength of cellulose-based materials.

**Fig. 5:**
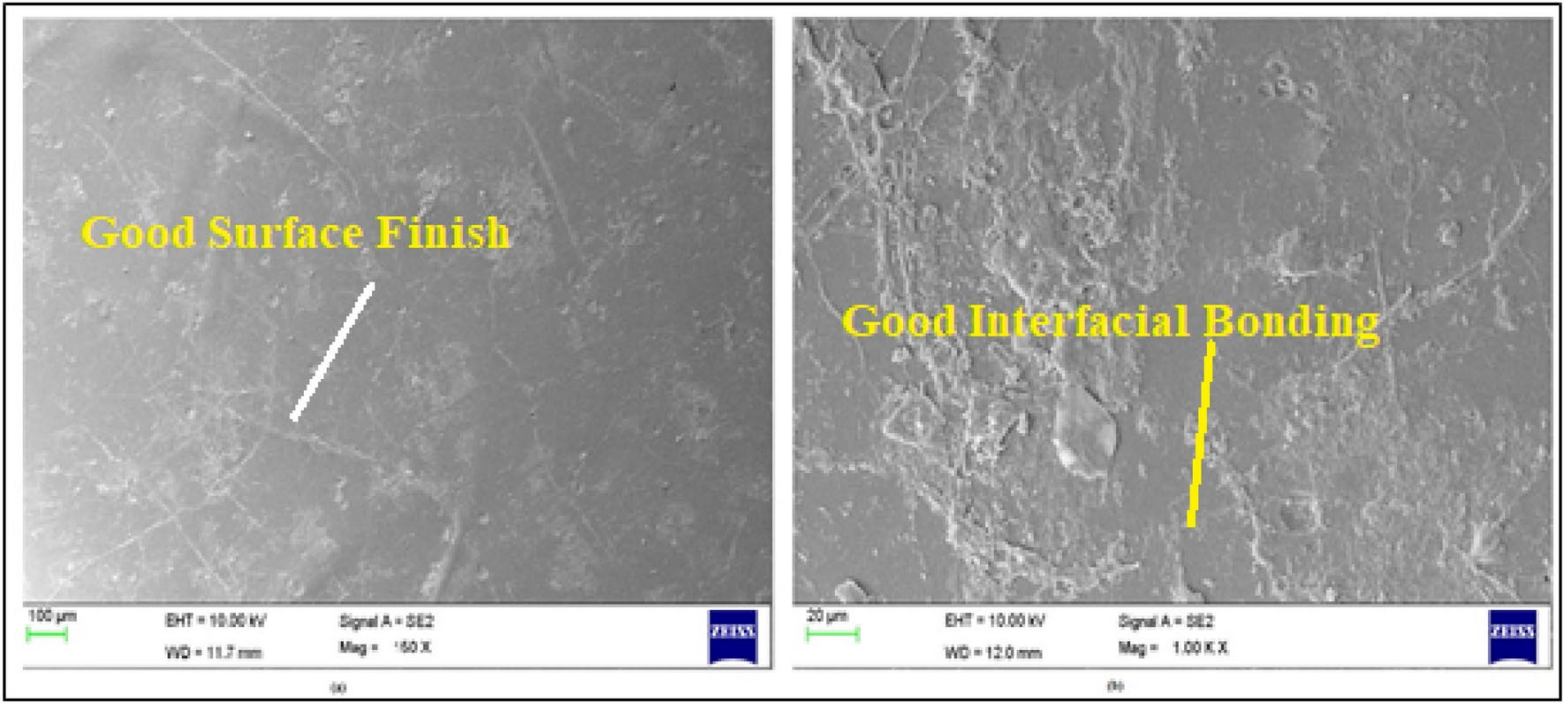
SEM image of the composite sample SM2.

**Fig. 6:**
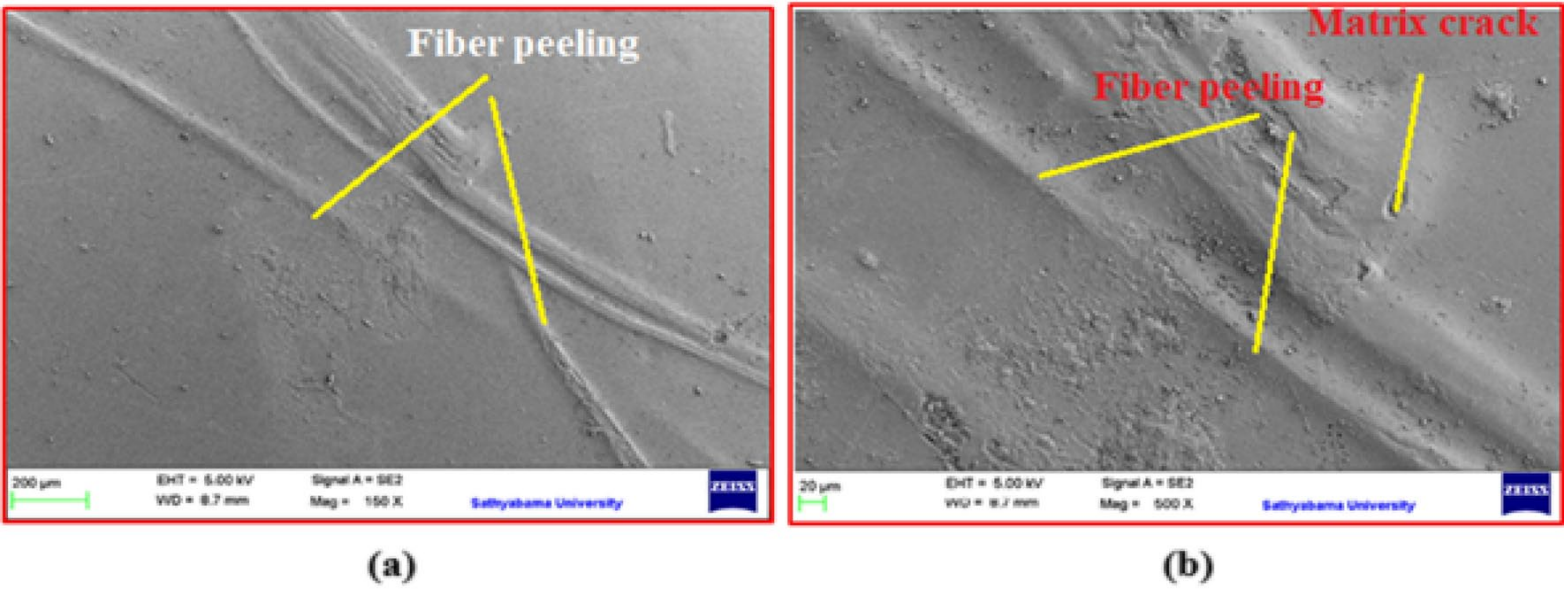
SEM images of the composite samples (**a**) SM1 and (**b**) SM3.

Considering that the OH groups present in the fibers, corresponding mostly to the alcoholic hydroxyls (weak acids), it can be proposed that the interaction was similar to what happens during a traditional mercerization treatment^21–23^. The SEM image of the fractured flexural sample SM2 is presented in Fig. 5. It was evident that better interfacial adhesion existed between the fiber and matrix, which resulted to better bonding and improvement in the properties, when compared with the fractured samples SM1 and SM3 (Fig. 6).

### Modal analysis

**Fig. 7:**
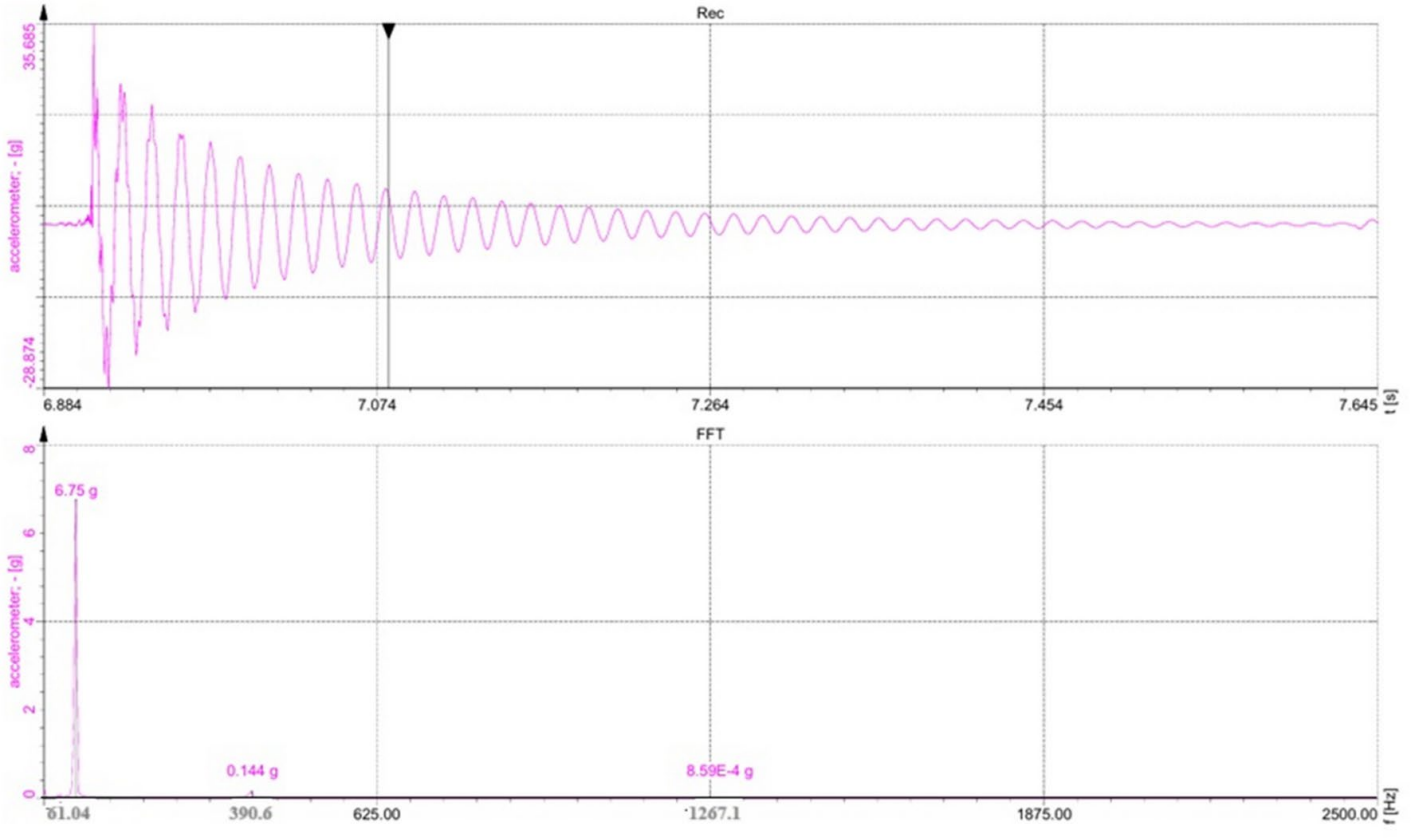
Amplitude *versus* frequency plot for composite sample SM2.

In this study, the dynamic characteristics of the composite structures were investigated by an experimental modal analysis, a technique used to determine the basic parameters for the dynamic behavior of a structure. In particular, the study aimed to determine the natural frequency and modal damping, which were crucial indicators of how the structure responded to dynamic forces. To achieve this, a modal analysis was performed using an impulse hammer test. In this method, the structure was excited with an impulse hammer and the resulting vibrations were analyzed to determine the essential information about the natural frequency and the associated modal damping. The FVT test was carried out on both treated and untreated composite samples under cantilever mode. A sample plot of amplitude *versus* frequency is shown in Fig. 7, the natural frequencies at modes I, II and mode III were taken from the peak of the curve.

Under mode I, among the various surface treated composites, the result established that composite samples SM1 and SM2 exhibited maximum values of natural frequencies of 56.15 and 61.04 Hz, respectively. Similarly, its modes II and III frequencies were 390 and 1267 Hz, respectively. The treatment with NaHCO_3_ resulted to better enhancement in natural frequency values when compared with both pure resin and other types of composite samples. Specifically, the treated composites exhibited a substantial 39% increase in natural frequency when compared with the pure resin. Additionally, in modes I, II and III, the treated composites demonstrated a remarkable increase, ranging from 18 to 69% in natural frequency values when compared with the other types of composites. The experimental results were compared with the theoretical values derived, using Eq. (2). Moreover, Table 3 presents the comparative analysis of the experimental and theoretical natural frequency values. It was noticeable that the theoretical natural frequency values slightly exceed the experimental values, which was due to certain underlying assumptions, such as ideal bond between fiber and matrix and uniform distribution of loads. It can be remarkably observed among the composites studied that composite sample SM2 exhibited the lowest damping factor of 0.0136 in mode I (Table 3). This was the lowest damping factor of all the composites. Notably, it indicated that the natural frequency of the composites increased with a decrease in damping factor, simultaneously. The NaHCO_3_ treatment mostly removed elements from the surface of fiber, such as pectin, lignin and waxy compounds. This treatment consequently improved the contact between the fiber and the matrix, leading to an increased in the stiffness of the composite. It also resulted in a more textured surface on the fiber, which promoted greater adhesion between the fiber and the matrix. As a result of these changes, the natural frequency of the composite was mainly determined by two factors: stiffness and the reciprocal of mass.

**Table 3:** Experimental and theoretical natural frequencies and damping factors of the composite samples.

Type of composite	Nomenclatures	Natural frequency (Hz)								Damping factor (ξ)		
		Experimental				Theoretical						
		Mode										
		I	II	III	I		II	III	I		II	III
Polyester resin	PR	13.43	119.6	390.6	20.75		121.5	221.3	0.0704		0.0568	0.0432
Untreated	UT	32.96	231.9	659.2	28.15		176.5	494.2	0.0333		0.0273	0.0315
NaOH	SM1	56.15	349.1	842.3	58.23		335.2	812.2	0.0148		0.0136	0.0125
NaHCO_3_	SM2	61.04	390.6	1267.1	63.23		382.2	928.2	0.0136		0.0129	0.0121
Cr_2_SO_4_	SM3	53.71	273.4	583.5	54.21		282.2	562.2	0.0158		0.0139	0.0128

As shown in Eq. (2) above, increasing the stiffness of a composite (e.g. by improving the fiber-matrix bond through surface texturing) will increase the natural frequency. In addition, reducing the bulk of the composite (e.g. by using lighter fibers or reducing the thickness of the material) can increase the natural frequency, but stiffness usually has a greater effect. The interaction between increased stiffness and a rougher surface texture synergistically increased the natural frequency of composite sample SM2. This phenomenon can be related to the SEM images in both Figs. 5 and 6, as similarly reported^8,10,24^. This effect can be attributed to the increased stiffness of the material and the efficient energy dissipation that occurs between the fiber and the matrix, which exceeds that of the polyester resin. In addition, a comparison of both damping and natural frequencies obtained from the present study on woven jute mat/polyester with similarly treated natural fiber reinforced composite samples are given in Table 4.

**Table 4:** Comparison of various similar treated natural fiber-reinforced composite samples.

Specimen code	Fiber/matrix	Surface modification type	Natural frequency (Hz)	Damping	Reference
Current work	Woven jute mat/polyester	UT	32.96	0.0333	
		SM1	56.15	0.0148	
		SM2	61.04	0.0136	
		SM3	53.71	0.0158	
1	Carbon and bamboo fiber/epoxy	Untreated	51.94	0.0164	^25^
		KMnO_4_	51.77	0.0197	
		NaOH	46.17	0.0220	
3	Flax fabric/epoxy	NaOH	16.83	1.370	^26^
	Linen fabric/epoxy		17.63	1.170	
4	Intra-ply hybrid banana/jute woven/polyester	NaOH	54.00	0.0569	^27^
		KMnO4	44.00	0.0534	
		Benzoyl chloride	49.00	0.0547	
5	Jute/epoxy	NaOH (1.0–7.5%)	35–45	–	^10^
6	*Phoenix* sp./epoxy	NaHCO_3_	UT	46.42	^11^
			24 h	48.00	
			120 h	51.68	
			240 h	48.00	

The current work has a higher natural frequency and lower damping factor compared to other types of treatments (Table 4). Due to these vibration properties, the composites can be used in sports equipment such as rackets, skis and bicycles, where vibration control is essential for improved comfort and performance, can use the improved composites for vibration damping. These composites can also provide lightweight protection with greater vibration absorption in products such as padding or helmets, reducing the risk of impact-related injuries.

### Contact angle measurement

**Fig. 8:**
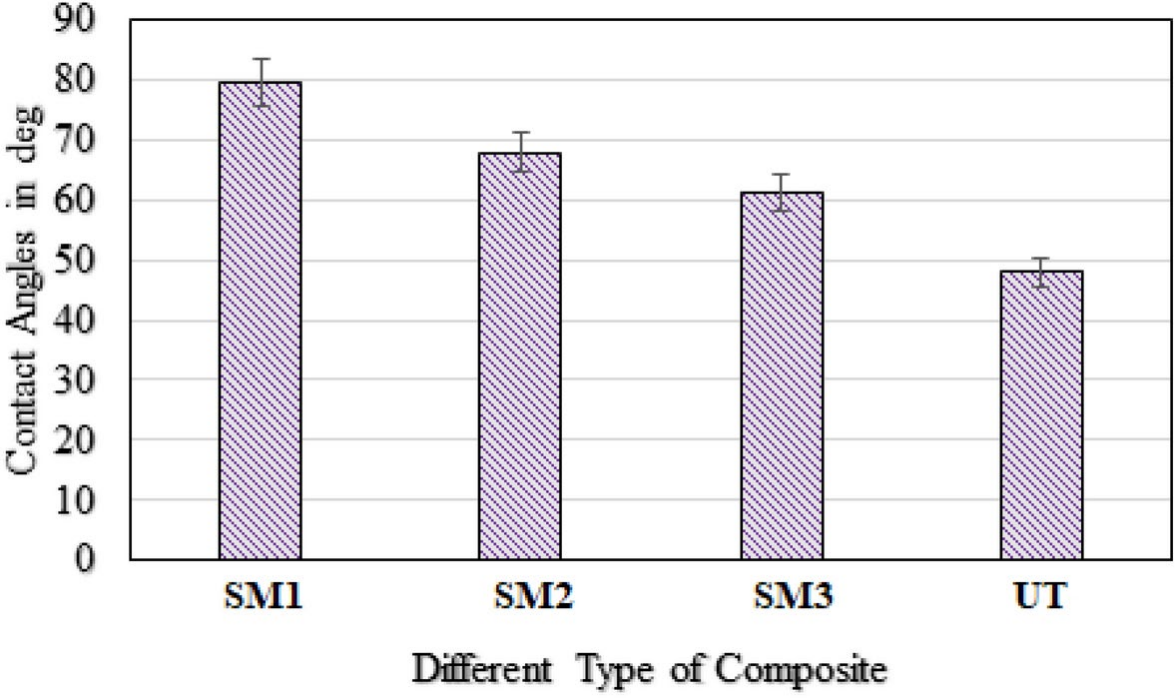
Wettability characteristics of both treated and untread the jute fiber samples.

**Fig. 9:**
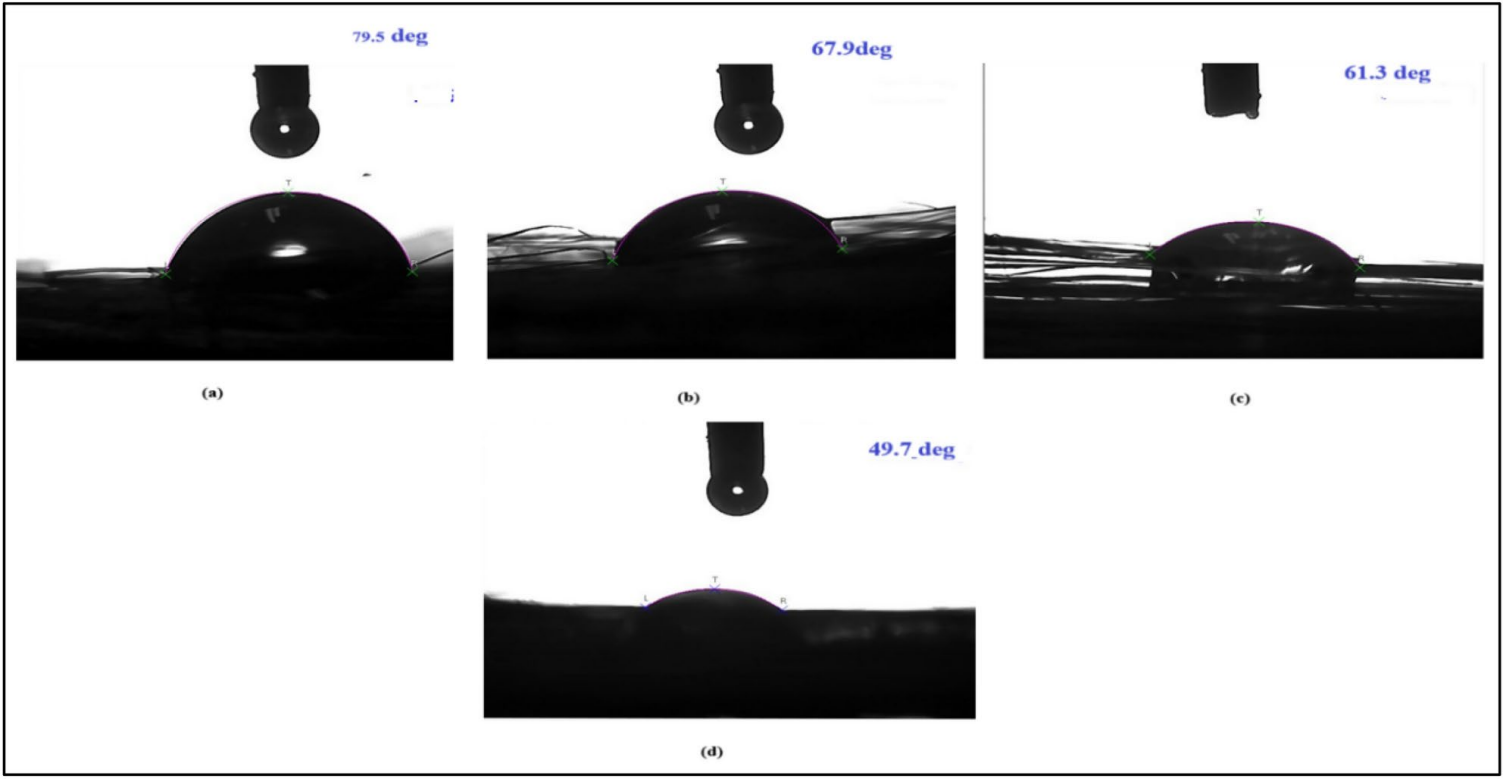
Measured contact angles (in degrees) of (**a**) NaOH, (**b**) NaHCO_3_, (**c**) Cr_2_SO_4_ treated and (**d**) untreated jute fiber/polyester resin samples.

The wettability properties of the untreated and treated fibers were calculated by using the contact angle measure analysis. Contact angle was measured by placing a drop of polyester resin on the untreated and treated jute fiber surfaces, which supported the determination of wettability. Wettability characteristics of both treated and untread the jute fiber samples are presented in Fig. 8. It was evident that the surface treating agent applied creates a difference in the interaction of fiber–resin. The contact angles with deviation in each value for treated and untreated jute fibers were measured with a contact angle meter. It was evident that Cr_2_SO_4_ treated sample lowest contact angle when compare with other treatments, which lied between 0 < ϴ < 90 degrees (Fig. 9). It showed that the fibers were partially wet by resin [28]. The result established that surface modification had considerable effect on contact angles. When a polyester resin drop was placed in contact with a jute fiber, it spread instantaneously towards its equilibrium shape, as when treated with NaOH, NaHCO_3_ and Cr_2_SO_4_ chemicals against the untreated fiber. In untreated jute fiber, the wettability between the non-polar resin molecules and presence of polar OH group on the untreated jute fiber curbed the spreading of resin, and it remained as a droplet on the fiber surface. According to other treated jute fiber, the contact angle of the resin drops rapidly decreased for Cr_2_SO_4_-treated jute fiber. In addition, the contact angle of a polyester resin drops applied to the surfaces of jute before and after treatment indicated that much resin absorption improved the following chemical treatment. Therefore, a greater adhesive bond to polyester resin was observed from the chemically treated surface, which indicated the cause of the interaction between the non-polar fiber surfaces and resin^29^.

### Sound absorption property

**Fig. 10:**
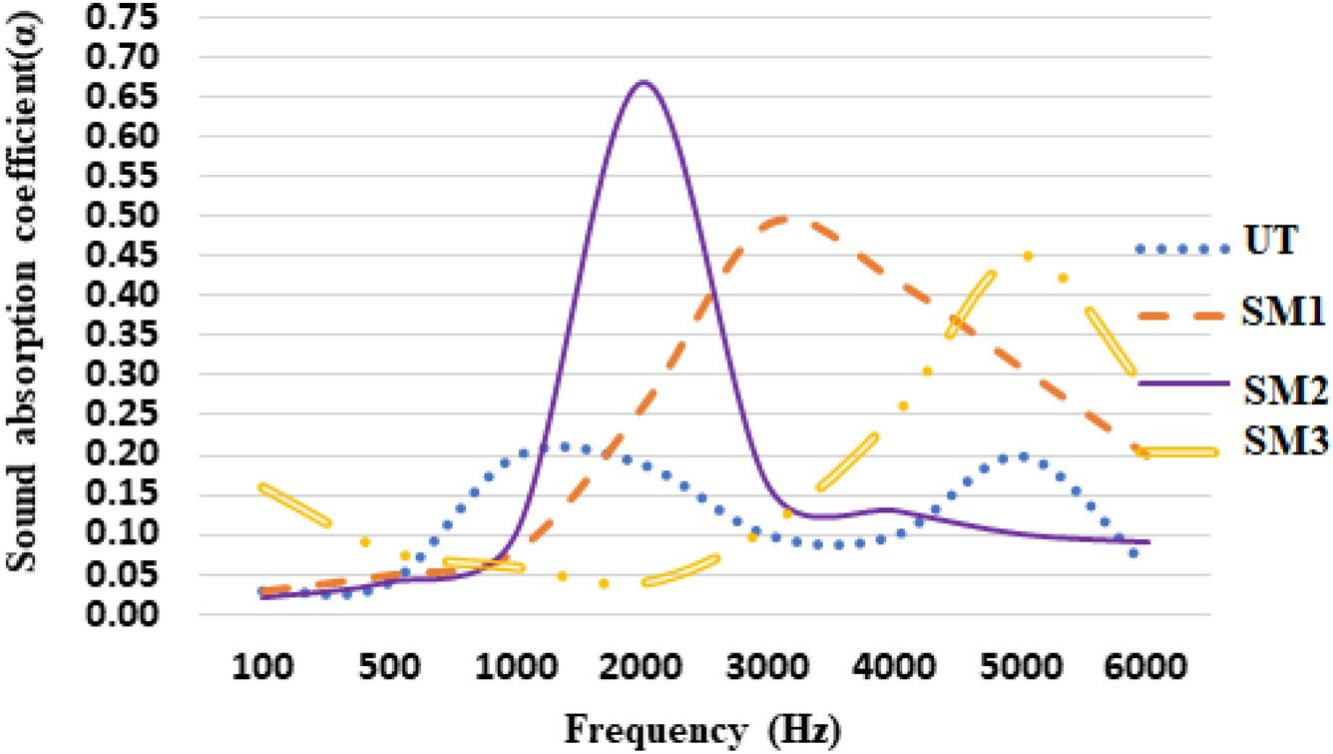
Sound absorption coefficients of the composite samples.

The acoustic behaviors of surface modified composites were determined, using large and tiny impedance tubes. The sound absorption coefficient (α) was calculated with aid of a mixture of smaller and larger tubes operating in the frequency range of 63–6300 Hz. Figure 10 depicts the sound absorption coefficients *versus* frequencies of the various composites.

Comparatively, composite sample SM2 recorded the highest sound absorption coefficient value of 0.67 at around 2000 Hz, which was 69% higher than that of untreated composite sample and around 29–72% higher than other treated composites. The surface modified fiber was free from unwanted impurities, including oil, wax, dust, pectin, lignin and others. Moreover, NaHCO_3_ treatment exhibited micropores on the fiber surface, which contributed to its highest sound absorption coefficient^17,21^. The composite sample SM3 exhibited lowest sound absorption coefficient among all the treated samples at all frequencies. The sound absorption coefficient increased, due to an effect of higher friction, increased energy loss of sound waves, size of the fiber, diameter and bulk density, better airflow resistivity and high viscous friction loss through vibration of air^19,20^. The phenomenon of sound absorption in natural fiber took these stages: when the sound wave first interacted with the fibers, heat was produced because of the viscous effects caused by both fibers and air cavities. Subsequently, this heat generation influenced the distribution of sound energy. Then, a constant process of heat addition occurred as a result of heat transfer interactions between various fibers, which helped to dissipate sound energy, as shown in past similar study^15^. Thirdly, the sound wave spread within a section of the fiber lumen and resulted to an internal vibration produced by air movement within the surrounding bulk materials. The absorption coefficient was used to determine the quantity of sound absorbed by the samples. In addition, Table 5 presents a comparison of the sound absorption coefficients of the jute straw/polyester woven composites produced in this study and the composite samples produced with bamboo/carbon fiber/epoxy and kenaf fiber in the literature.

**Table 5:** Comparative overview of acoustic characteristics of some fiber-based composites.

Specimen no	Fiber/matrix	Surface modification type	Sound absorption coefficient (α)	Reference
Current work	Woven jute mat/polyester	UT	0.21	–
		SM1	0.49	
		SM2	0.67	
		SM3	0.45	
1	Bamboo/carbon fiber/epoxy	Untreated	0.55	^25^
		KMnO_4_	0.58	
		NaOH	0.68	
2	Kenaf fiber	Untreated	0.562	^17^

### Theoretical prediction of sound absorption coefficient

**Fig. 11:**
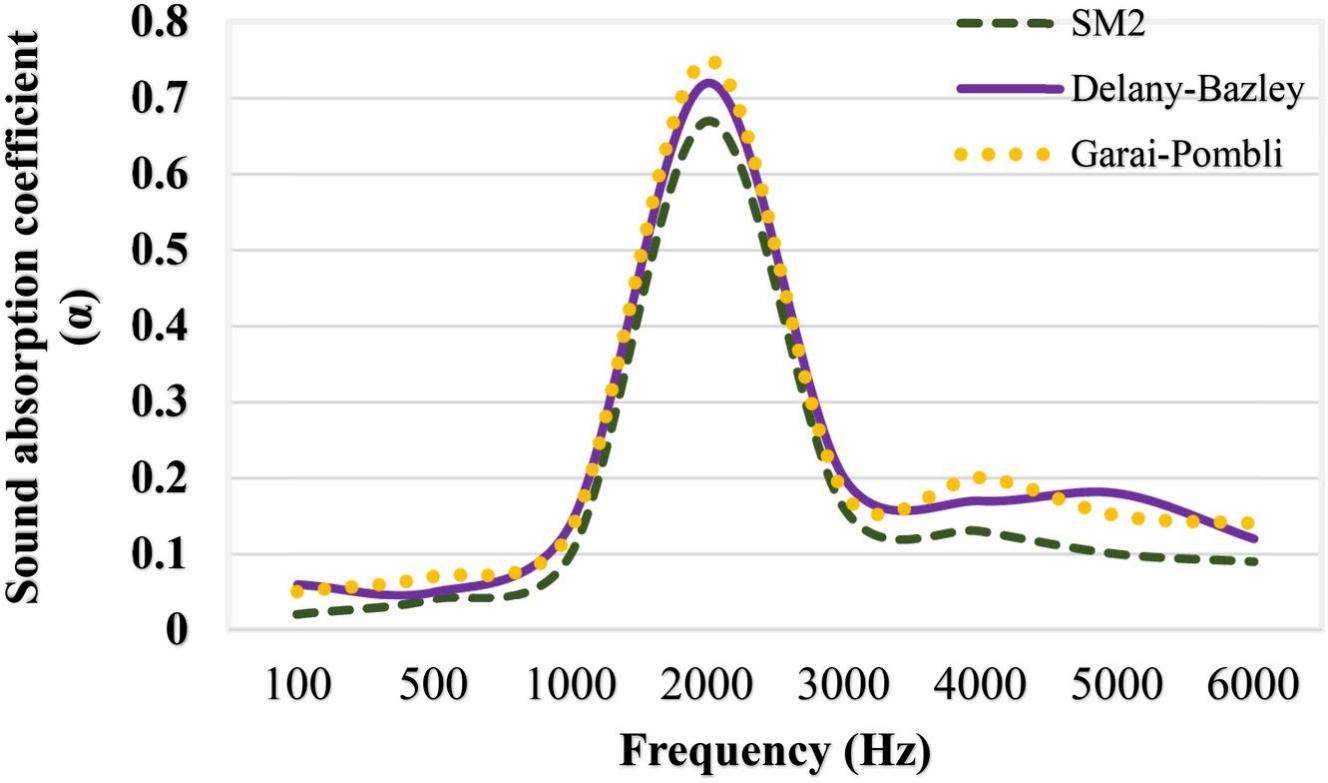
Theoretical sound absorption coefficients.

This study employed both Delany-Bazley and the Garai Pompoli models to determine the acoustic impedance (Zc) and propagation constant (Kc). These equations were employed to derive the coefficient of reflection. From the equations, ρ0 and C represent the density and speed of the air medium respectively, while σ and f denote the flow resistivity and frequency (ω = 2πf), respectively. From Fig. 11, the empirical results of the Delay-Bazley and Garai-Pompoli models were compared with the experimental data from the optimum composite sample SM2.

At 2000 Hz, the absorption coefficients of the empirical model were in good agreement with the experimental result, with a value of 0.67. Notably, the Garai-Pompoli and Delay-Bazley models yielded higher values, 0.72 and 0.75 respectively, highlighting the superior sound absorption properties of the surface modified composite. This was confirmed by the coefficient of sound absorption coefficient of 0.67 at 2000 Hz. The experimental data deviated by about 7 to 10% from the theoretical models originally developed for very thin fibers of less than 10 µm diameter. The jute fibers used in this study had bigger diameters; ranging from 50 to 60 µm. This difference would have contributed to the small disparities between theoretical and experimental predictions. It is important to note that the theoretical and experimental results exhibited a closer alignment at mid-frequency levels, which can be attributed to the assumption of uniform and homogeneous materials. This convergence is commonly notable between the results of the theoretical models and the experimental findings at these specific frequency ranges^23^. Therefore, these types of composites can be suggested for use in dashboards, flooring, door panels and automotive interiors, where noise and vibration damping can reduce road noise, increase comfort and improve the overall acoustic performance of the vehicle. A comparison of the physical and mechanical properties of untreated and alkali-treated jute fibers is shown in Table 6. Alkali-treated jute fibers are likely to be stiffer, have a higher natural frequency, a lower damping factor and possibly a lower sound absorption coefficient than untreated jute fibers. Alkali-treated fibers are therefore more suitable for applications where strength and stiffness are essential, but less suitable for applications where high energy dissipation or sound absorption is required.

**Table 6:** Comparison of the alkali treated and untreated jute fibers.

Fiber treatment	Density (g/cm^3^)	Diameter (µm)	Tensile strength (Mpa)	Young modulus (GPa)	Aspect ratio (L/D)	Reference
Untreated jute fiber	1.48	48	295	30	104	^31^
Alkali treated jute fiber	1.3	32	480	38	156	^31,32^

### Fractoscopy study

**Fig. 12:**
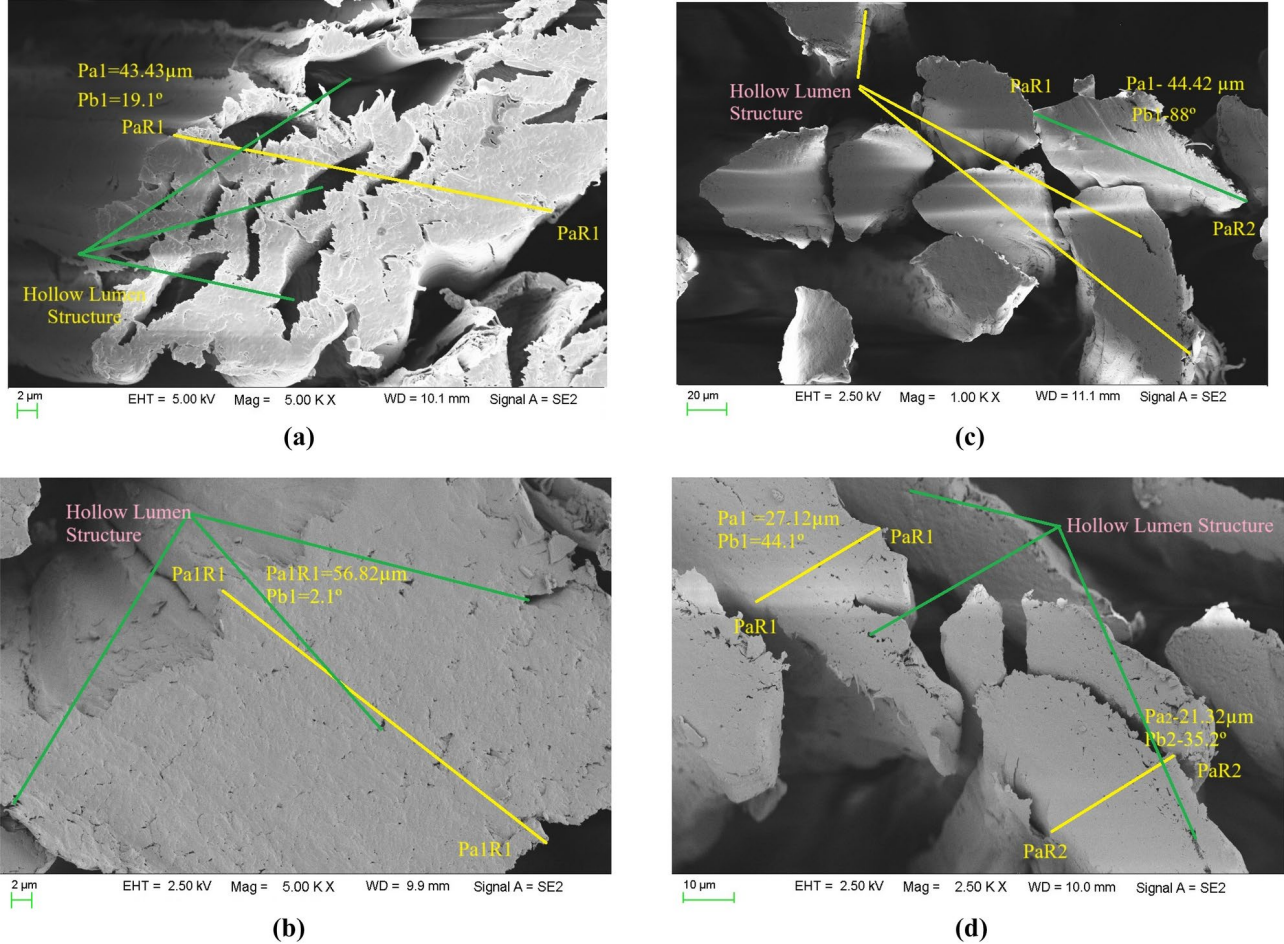
The SEM images of the (**a**) untreated, (**b**) Cr_2_SO_4_ (**c**) NaOH, and (**d**) NaHCO_3_ treated jute fibers.

The SEM images of untreated and NaOH, NaHCO_3_ and Cr_2_SO_4_ treated jute fibers are presented in Fig. 12a–d, respectively. The treated jute fibers had smaller hollow lumen structure, rougher fiber surface and thicker fiber wall when compared with the untreated jute fiber. In addition, the treated fibers contained less air cavities, lowering the air flow resistance^30^. Furthermore, the thicker fiber wall had fewer nano-fibrils, which reduced the impact of sound. NaHCO_3_ treated jute fibers exhibited more porous structure than other treatments. The sound absorption behavior was determined by the interconnected porous structure of the fiber materials and the thin wall surrounding by the nano-fibrils^31,33^.

## Conclusion

This study investigated the effect of surface modifications on the acoustic and vibration behaviour of the untreated and treated woven jute mat/polyester composites. Based on the consistent experimental and theoretical results obtained, the following conclusions were drawn:

- The surface modification of the fiber improved the natural frequency, flexural/vibration and acoustic behaviors of the composites. In addition, analysis through the SEM images highlighted that the application of the environmentally friendly sodium bi-carbonate treatment notably enhanced the interfacial adhesion bond between the fiber and the matrix.
- Chemical treatments can affect the damping factors of composites. NaOH treatment increased damping by reducing friction, while NaHCO_3_ and Cr_2_SO_4_ treatments improved molecular stability, allowing better energy storage. These treatments also caused microstructural changes, such as fiber-matrix interactions, which explain the observed dynamic behavior.
- Evidently, NaHCO_3_ treated composite sample SM2 exhibited the greatest sound absorption coefficient of 0.67 at around 2 kHz, which was 69% higher than that of the untreated composite samples and around 29–72% higher than other treated SM1 and SM3 counterparts. Summarily, the surface modification of the composite improved its vibro-acoustic properties for sustainable applications of nanomaterials and chemical treatments, to improve the interaction between fibers and the matrix. Eco-friendly treatments and hybrid composites will enhance performance even further while advancing sustainability. Smart composites and other multipurpose designs, along with improvements in manufacturing techniques, can offer customized vibration damping and noise reduction options. Furthermore, low-cost and high-volume production techniques will contribute to the commercial viability of these improved composites for a range of uses.

## Data Availability

The data that support the findings of this study are available from the corresponding author upon reasonable request.
